# Exercise and the dipeptidyl‐peptidase IV inhibitor sitagliptin do not improve beta‐cell function and glucose homeostasis in long‐lasting type 1 diabetes—A randomised open‐label study

**DOI:** 10.1002/edm2.75

**Published:** 2019-05-23

**Authors:** Eleonora Seelig, Beckey Trinh, Henner Hanssen, Arno Schmid‐Trucksäss, Helga Ellingsgaard, Mirjam Christ‐Crain, Marc Y. Donath

**Affiliations:** ^1^ Clinic of Endocrinology, Diabetes and Metabolism University Hospital Basel Basel Switzerland; ^2^ Department of Biomedicine University of Basel Basel Switzerland; ^3^ University of Cambridge Metabolic Research Laboratories, Wellcome Trust‐Medical Research Council Institute of Metabolic Science Addenbrooke's Hospital Cambridge UK; ^4^ Department of Sports Medicine, Institute of Exercise and Health Sciences University of Basel Basel Switzerland; ^5^ The Centre of Inflammation and Metabolism and the Centre for Physical Activity Research, Rigshospitalet University of Copenhagen Copenhagen Denmark

**Keywords:** beta‐cell regeneration, dipeptidyl‐peptidase IV inhibitor, exercise, glucagon‐like peptide‐1, interleukin 6, type 1 diabetes mellitus

## Abstract

**Background:**

Increasing evidence points to beta‐cell regeneration in individuals with type 1 diabetes mellitus (type 1 DM) at all stages of the disease. Exercise and glucagon‐like peptide‐1 (GLP‐1) independently improve beta‐cell function and glucose homeostasis in animal studies and in clinical trials in individuals with type 2 diabetes mellitus (type 2 DM). Whether a combination of both, exercise and GLP‐1, induces a similar effect in individuals with long‐lasting type 1 DM remains to be investigated.

**Methods:**

In an open‐label study, participants with long‐standing type 1 DM were randomly assigned to oral sitagliptin 100 mg daily for 12 weeks in combination with or without an exercise intervention. The primary end‐point was change in the area under the concentration‐time curve of C‐peptide during a mixed meal tolerance test before and after 12 weeks of intervention.

**Results:**

A total of 24 participants were included in the study and treated with sitagliptin, 12 participants were allocated to a 12‐week exercise intervention. After 12 weeks, there was no difference in the change of AUC C‐peptide between groups (exercise: 0 [−1424 to 1870], no exercise: 2091 [283‐17 434]; *P* = 0.09). HDL improved in the exercise intervention group compared to the group with sitagliptin only (exercise: 0.11 [−0.09 to 0.27]; no exercise: −0.18 [−0.24 to 0.01]; *P* = 0.04). AUC glucose was numerically slightly lower in the exercise intervention group but this did not translate into changes in HbA1c.

**Conclusion:**

The combination of exercise and sitagliptin had no effect on beta‐cell function in individuals with long‐lasting type 1 DM.

## 1 INTRODUCTION

Type 1 diabetes mellitus (type 1 DM) is characterized by an autoimmune mediated process which leads to the destruction of insulin‐producing beta cells.^1^ Hyperglycaemia develops as a consequence of the gradual loss of beta cells. Until recently, it was assumed that all insulin‐producing cells are being destroyed over the course of the disease. Our group and others have shown that even years after diagnosis of type 1 DM, viable beta cells are present and able to secrete at least low amounts of insulin.^2^, ^3^ Since residual beta‐cell function prevents complications such as retinopathy, nephropathy and hypoglycaemia,^4^ it is of great interest to preserve and enhance the function of these remaining insulin‐producing cells.

Whether beta‐cell replication exists in adults is still controversial.^5^, ^6^ Increasing evidence suggests that beta‐cell differentiation and regeneration occur throughout the course of the disease.^7^, ^8^, ^9^ Several hormones have been implicated in this process, in particular the gut hormone glucagon‐like peptide‐1 (GLP‐1).^10^ GLP‐1 is acutely released from intestinal L cells in response to nutrient ingestion and from pancreatic alpha cells, especially during metabolic stress^11^, ^12^, ^13^ and improves glucose homeostasis. Drugs such as dipeptidyl‐peptidase IV (DPP‐IV) inhibitors, which inhibit the degradation of GLP‐1, are widely used for treatment of type 2 diabetes mellitus (type 2 DM).^14^

Similar to GLP‐1, physical exercise is known to ameliorate glucose control in type 2 DM.^15^, ^16^ We have previously shown that GLP‐1 plays an important role in the exercise‐induced improvement of glucose homeostasis in mice.^17^ Physical exercise led to release of interleukin‐6 (IL‐6) from skeletal muscle, which triggered GLP‐1 secretion and subsequently led to an improvement in insulin secretion and glucose homeostasis. Based on these findings, we hypothesized that in individuals with type 1 DM, residual beta‐cell function would improve with exercise via IL‐6‐dependent up‐regulation of GLP‐1. We further explored whether the combination of exercise and treatment with a DPP‐IV inhibitor would amplify this endocrine loop in people with long‐lasting type 1 DM.

## 2 MATERIALS AND METHODS

### 2.1 Study design and participants

Between November 2013 and August 2016, 24 participants with type 1 DM were included in this randomized, open‐label study. The study was conducted at the University Hospital Basel.

Participants were eligible if they aged 18‐58 years, were diagnosed with type 1 DM according to American Diabetes Association criteria of more than 2 years, had positive glutamic acid decarboxylase and/or islet antigen‐2 autoantibodies, were well‐controlled (HbA1c < 63.9 mmol/mol resp. <8%), were on a stable treatment for the last 3 months and had a body mass index (BMI) between 18 and 30 kg/m^2^. Due to the slow recruitment process, inclusion criteria for HbA1c and BMI were extended during the study: the range of HbA1c was increased from 7.5% to 8%, and the upper limit of BMI was increased from 28 to 30 kg/m^2^. Regular physical activity of more than 4 hours per week, independent of the intensity of exercise, was an exclusion criterion. Other exclusion criteria were any inflammatory, infectious or immunosuppressive disease, any immunosuppressive treatment, pregnancy or breastfeeding, history or signs of cardiovascular disease, proliferative retinopathy, nephropathy or neuropathy. Patients were recruited by advertisement from the outpatient clinic of the University Hospital Basel and from outpatient diabetes clinics in the north‐western part of Switzerland. All participants provided written informed consent.

The study was approved by the regional ethical committee (EKBB 349/12) and Swissmedic, and was conducted in accordance with the guidelines for Good Clinical Practice and the Declaration of Helsinki. The trial was registered at Clinicaltrials.gov NCT02127047.

### 2.2 Randomization and treatment

All participants received sitagliptin (Januvia^®^; MSD Merck Sharp & Dohme AG) 100 mg per os (po) once daily and were randomized in a 1:1 ratio to complete a 12‐week exercise intervention or to continue physical activity on their pre‐existent level. Randomization was performed by an external statistician.

### 2.3 Study assessment

All participants had a screening visit and four study visits. At the first study visit, a standardized mixed meal tolerance test (MMTT) with 360 mL of Boost^®^ containing 62 g carbohydrates, 15 g protein and 6 g fat (Nestle) was performed after an overnight fast. Baseline blood samples were taken immediately before ingestion of the mixed meal and 15, 30, 90 and 120 minutes afterwards. After the MMTT, a bicycle ergometer test was performed to assess VO_2_ max and determine the exercise load for the exercise intervention group. Participants of the exercise intervention were given instructions for an unsupervised bicycle training consisting of 5‐minute warm‐up followed by 45 minutes at 75% VO_2_ max and 5‐minute cool‐down for at least three times a week on top of their pre‐existent physical activity level. Heart rate monitors (Polar Watch; Polar) were dispensed to participants in the intervention group to be worn during each exercise session. Adherence to the exercise target was evaluated by the read‐outs of the heart rate monitor. Daily used insulin dose at baseline and after the intervention was documented during 3 days with insulin diaries. Sitagliptin treatment was initiated after completion of the MMTT and VO_2 _max test on the first day of the study. All participants were instructed to take one tablet of sitagliptin once a day. After 4 and 8 weeks, participants returned to the research facilities for assessment of safety including the occurrence of hypoglycaemia and compliance. After 12 weeks and 24 hours after the last dose of sitagliptin, the 2‐hour MMTT and the bicycle ergometer test were repeated.

C‐peptide and insulin were measured using Elecsys 2010 (Roche Diagnostics). Total GLP‐1 was assessed with NL‐ELISA (Mercodia). Glucose, HbA1c, high‐sensitivity C‐reactive protein (hsCRP) and lipids were measured at the routine laboratory, Department of Clinical Chemistry, University Hospital Basel, Switzerland. The reported HOMA index was calculated according to Matthews et al.^18^

### 2.4 Study end‐points

The primary end‐point was change in the area under the concentration‐time curve (AUC) of C‐peptide during a MMTT at baseline and after 12 weeks with or without an exercise intervention. Predefined secondary end‐points were change in glucose, HbA1c, insulin sensitivity, insulin requirements, total GLP‐1, change in lipids profile, hsCRP, creatine kinase (CK).

### 2.5 Statistical analysis

The sample size of 12 participants per group was based on the assumption of a 30% change in beta‐cell function in response to a physiological stimulus as compared to baseline, providing 90% power and *P* < 0.05.

The primary analysis followed the intention to treat principle, that is, participants with complete follow‐up were analysed in the groups to which they were randomized. Discrete variables are expressed as counts (percentages) and continuous variables as median (interquartile range [IQR]). The Mann‐Whitney *U* test was used for continuous data and the Fisher exact test for categorical data to compare changes across treatment groups. The Wilcoxon paired signed‐rank test was used for comparisons within subjects. The AUC C‐peptide over 120 minutes during the MMTT was calculated using the trapezoid rule. *P*‐value < 0.05 was defined as significant. Data were analysed using GraphPad Prism Vers 7 (GraphPad Software Inc).

## 3 RESULTS

**Figure 1:**
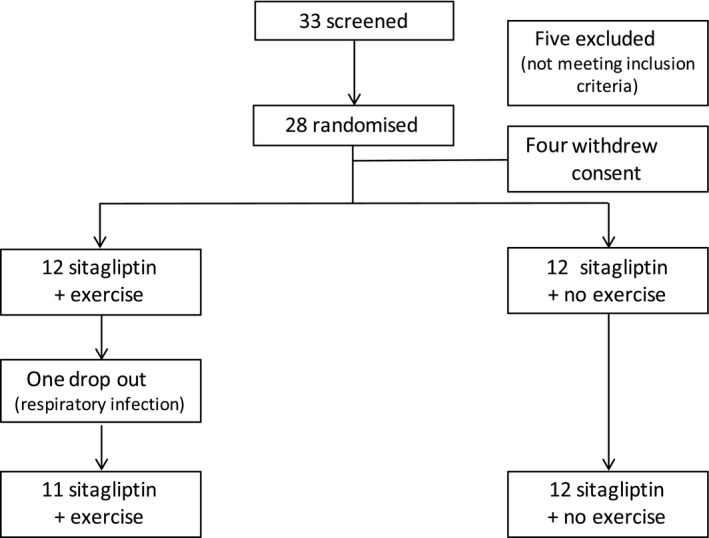
Enrolment

A total of 33 individuals were screened, five did not meet inclusion or exclusion criteria. Twenty‐eight people were enrolled in the study, and four withdrew their consent. One participant in the exercise intervention group dropped out after randomization due to a prolonged upper respiratory tract infection. Twenty‐three participants completed the study (Figure 1). Baseline characteristics were similar in both groups, apart from VO_2_ max, which was slightly higher in the exercise group (Table 1).

**Table 1:** Comparison of baseline characteristics between treatment groups

	Exercise	No exercise	*P*‐value
Age (y)	33.18 (27.07‐38.57)	35.08 (27.52‐45.37)	0.47
Sex			
Female, n (%)	4 (25%)	3 (33.33%)	>0.99
Male, n (%)	8 (75%)	9 (66.67%)	0.68
Body mass index (kg/m^2^)	25.35 (24.03‐27.85)	25.2 (24.03‐27.85)	0.54
Diabetes duration (y)	15.69 (9.26‐21.01)	8.82 (4.60‐14.48)	0.10
Type 1‐associated antibodies			
Glutamic acid decarboxylase antibodies positive, n (%)	7 (58.3%)	8 (66.7%)	>0.99
Islet antigen‐2 antibodies positive, n (%)	7 (58.3%)	5 (41.7%)	0.68
HbA1c (mmol/mol)	57 (53‐61)	55 (50‐61)	0.31
HbA1c (%)	7.4 (7.02‐7.7)	7.15 (6.72‐7.7)	
Fasting C‐peptide (pmol/L)	16.5 (2.9‐30)	187.5 (9.5‐324.8)	0.11
AUC C‐peptide	403 (348‐7589)	4032 (348‐59 389)	0.17
Fasting Insulin (pmol/L)	4.45 (1.3‐15.7)	9.75 (5.82‐24.25)	0.18
Daily long‐acting insulin dose (units/d)	16 (13.03‐25.5)	15.55 (13.25‐26.63)	0.70
High‐sensitive C‐reactive protein (mg/L)	0.74 (0.30‐1.75)	0.74 (0.29‐1.42)	>0.99
Total cholesterol (mmol/L)	4.07 (3.38‐4.69)	4.38 (4.13‐4.43)	0.47
Low‐density lipoprotein (mmol/L)	2.07 (1.63‐2.40)	2.45 (2.19‐2.73)	0.13
High‐density lipoprotein (mmol/L)	1.77 (1.49‐2.01)	1.38 (1.25‐1.86)	0.09
Triglycerides (mmol/L)	0.63 (0.41‐0.83)	0.68 (0.57‐0.81)	0.52
VO_2 _max (L/min/kg)	0.037 (0.033‐0.045)	0.031 (0.027‐0.035)	0.03

Note

The Mann‐Whitney *U* test was used for continuous data and the Fisher exact test for categorical data to compare treatment groups; data represent median values with interquartile ranges.

**Figure 2:**
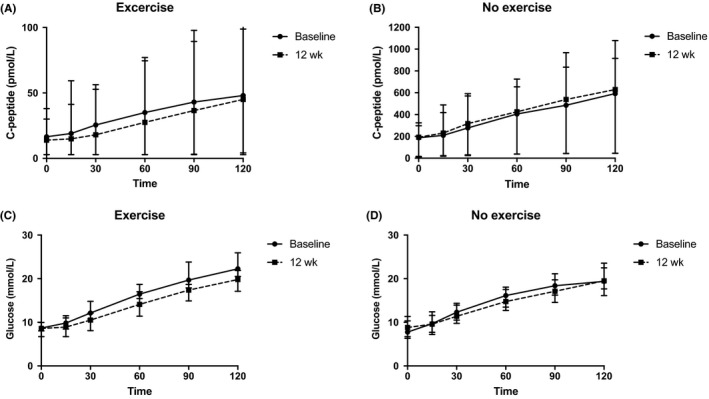
C‐peptide during 2‐h mixed meal tolerance test (MMTT) at baseline and after 12 wk in (A) patients with sitagliptin and exercise intervention and (B) patients with sitagliptin only. Glucose levels during 2‐h MMTT at baseline and after 12 wk in (C) patients with sitagliptin and exercise intervention and (D) patients with sitagliptin only. Data represent median and interquartile range

**Figure 3:**
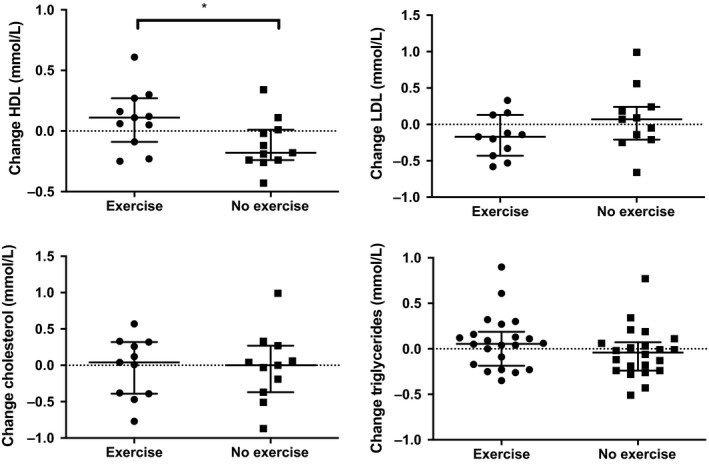
Change in lipid levels according to intervention. Data represent median and interquartile range

After a 12‐week intervention, there was no difference in the change of AUC C‐peptide between groups (*P* = 0.09) (Table 2, Figure 2). Interestingly, there was an increase in AUC C‐peptide after a 12‐week treatment period as compared to baseline in the group with sitagliptin only, but it did not reach statistical significance (*P* = 0.07, Figure S1). There was no change in AUC glucose between the two groups (*P* = 0.23) (Figure 2). Change in fasting glucose (*P* = 0.52), HbA1c (*P* = 0.79), insulin sensitivity (*P* = 0.45), daily insulin use (*P* = 0.32), AUC total GLP‐1 (*P* = 0.78) did not differ between groups (Table 2). hsCRP was similar in both groups (*P* = 0.73). Interestingly, high‐density lipoprotein cholesterol (HDL‐C) improved in the exercise intervention group (*P* = 0.04), while change in total cholesterol (*P* = 0.83), low‐density lipoprotein cholesterol (LDL‐C) (*P* = 0.19) and triglycerides (*P* = 0.11) remained similar between groups (Table 2, Figure 3).

**Table 2:** Comparison of change before and after 12 wk intervention between treatment groups

	Exercise	No exercise	*P*‐value
AUC C‐peptide	0 (−1424 to 1870)	2091 (283‐17 434)	0.09
AUC glucose	−295 (−567 to 90)	−47.5 (−351 to 223)	0.23
Fasting glucose (mmol/L)	−1.55 (−3.47 to 2)	−0.5 (−2.95 to 1.32)	0.52
HbA1c (mmol/mol)	−3 (−4 to −1)	−2 (−5 to −1)	0.79
HbA1c (%)	−0.3 (−0.4 to 0.1)	−0.2 (−0.5 to 0.1)	
Insulin sensitivity (HOMA Index)	0.05 (−0.3 to 0.275)	0.0 (−0.42 to 0.07)	0.45
AUC total glucagon‐like peptide‐1	−35.6 (−115.1 to 17.8)	−29.5 (−125.7 to 23.95)	0.78
Average long‐acting insulin use (units/d)	0 (−0.32 to 0.35)	0 (−0.19 to 1)	0.32
High‐sensitivity C‐reactive protein (mg/L)	−0.1 (−0.42 to 0.26)	−0.07 (−0.28 to 0.09)	0.73
Total cholesterol (mmol/L)	0.04 (−0.39 to 0.32)	0.0 (−0.37 to 0.27)	0.83
Low‐density lipoprotein (mmol/L)	−0.17 (−0.43 to 0.13)	0.07 (−0.21 to 0.24)	0.19
High‐density lipoprotein (mmol/L)	0.11 (−0.09 to 0.27)	−0.18 (−0.24 to 0.01)	0.04
Triglycerides (mmol/L)	0.05 (−0.18 to 0.18)	−0.04 (−0.24 to 0.07)	0.11
Creatine kinase (U/L)	29 (−1 to 69)	2 (−17.25 to 18)	0.17
Weight (kg)	−1.2 (−3 to −0.4)	−0.45 (−1.8 to 1.275)	0.15
Systolic blood pressure (mm Hg)	2 (−6 to 6)	2 (−5 to 3.5)	0.77
Diastolic blood pressure (mm Hg)	1 (−11 to 10)	−2 (−12.5 to 1)	0.27
Resting heart rate (beats/min)	−2 (−8 to 8)	3 (−4.5 to 8)	0.42
VO_2_ max (L/min/kg)	0.001 (−0.002 to 0.001)	−0.0004 (−0.002 to 0.003)	0.40

Note

Mann‐Whitney *U* test was used to compare change across groups; data represent median values with interquartile ranges.

Weight decreased in both groups, and the difference between groups was not significant (Table 2). Systolic and diastolic blood pressure as well as heart rate did not change in both groups (Table 2).

Change in CK tended to be higher in the exercise intervention group, but did not reach significance (*P* = 0.17) (Table 2).

Change in VO_2_ max was not different between groups (*P* = 0.97) (Table 2). Training compliance in the exercise intervention group was as follows: four participants (36.3%) completed an average of ≥3 sessions per week, 5 (45.4%) completed at least 2‐2.9 sessions per week, 2 (18.1%) trained 1‐1.9 times per week. Seven participants (64%) accomplished at least 50% of the training sessions at 75% of VO_2_ max during for at least 25 minutes of training.

The number of adverse events was similar in both groups (Table 3). The most frequent adverse event was a common cold (seven adverse events in each group).

**Table 3:** Adverse events

Adverse event, n (%)	Exercise (n = 12)	No exercise (n = 12)
Adverse events	17 (48.6%)	18 (51.4%)
Drug related	0 (0%)	0 (0%)
Exercise related	0 (0%)	0 (0%)
Serious adverse events	0 (0%)	0 (0%)
Maximum severity of adverse events		
Mild	15 (42.8%)	16 (45.7%)
Moderate	1 (2.8%)	2 (5.7%)
Severe	1 (2.8%)	0 (0%)
Adverse events leading to withdrawal	1 (2.8%)	0 (0%)

## 4 DISCUSSION

This is the first randomized trial to study the role of exercise in combination with the DPP‐IV inhibitor sitagliptin in individuals with long‐standing type 1 DM.

We found no improvement in beta‐cell function with exercise and sitagliptin or sitagliptin alone after a 12‐week study period. This finding contrasts with animal models of type 1 DM as well as clinical studies in type 2 DM and healthy individuals where exercise and DPP‐IV inhibitors independently enhanced beta‐cell function.

In rat models of type 1 DM, for example, physical exercise boosted beta‐cell proliferation as well as cell mass after near total loss of pancreatic tissue.^19^, ^20^ Similar results were obtained in rat models of type 2 DM, where exercise led to an increase in beta‐cell mass.^21^, ^22^, ^23^ In line with these findings, clinical studies in individuals with type 2 DM and healthy people showed an improvement of beta‐cell function with exercise.^24^, ^25^ Nonetheless, in individuals with newly diagnosed type 1 DM, a 12‐month exercise training did not result in enhanced beta‐cell function.^26^

Similar to exercise, GLP‐1 improved beta‐cell function in animal models of type 1 DM. Indeed both, GLP‐1 receptor agonists and DPP‐IV inhibitors were shown to induce beta‐cell proliferation and reverse new‐onset diabetes.^27^, ^28^, ^29^ Still, in individuals with type 1 DM, treatment with GLP‐1 receptor agonist or DPP‐IV inhibitors yielded more heterogeneous results with some studies showing a beneficial effect on glucose control and other with no effect at all.^30^ There may be several reasons why results from animal models of type 1 DM and people with type 2 DM do not convincingly translate to individuals with type 1 DM. First, the ongoing autoimmune process could prevent a significant improvement of beta‐cell function. Second, a critical mass of beta cells may be necessary to produce a measurable improvement, which could have been especially critical in our cohort of individuals with long‐lasting disease. While there was no statistical difference in residual beta‐cell function between groups at baseline, there was a trend towards lower values in the exercise group. As a solid C‐peptide response may be critical for detecting a difference in beta‐cell function, a clinical trial in patients with newly diagnosed type 1 DM with substantial residual beta‐cell function may provide different results. Third, over the course of the disease, beta cells may develop impaired GLP‐1 signalling and therefore could become unresponsive to GLP‐1.^31^ Further studies are needed to understand these underlying mechanisms. In our study, peak oxygen consumption at baseline was slightly higher in the exercise intervention group and did not improve significantly during the exercise training. It therefore could be that a more intense exercise programme in combination with a more potent GLP‐1 receptor agonist would yield different results. Another limitation is the small sample size of this exploratory study. Future studies with bigger sample sizes may detect a difference between the groups.

High‐density lipoprotein levels significantly increased in participants with type 1 DM who had both exercise and sitagliptin. Regular exercise was shown to improve HDL in nondiabetic people as well as in individuals with type 1 DM.^32^, ^33^ Similarly, a single dose of oral sitagliptin reduced postprandial lipidemia in healthy volunteers.^34^ Low HDL levels are a risk marker for cardiovascular disease,^35^ which has become the leading cause of death in people with type 1 DM above age 30 years.^36^ Whether exercise in combination with sitagliptin can reduce cardiovascular disease in type 1 DM remains to be investigated.

In summary, we could not reproduce the findings of studies in animal models and people with type 2 DM with sitagliptin and exercise for improving beta‐cell function in individuals with long‐lasting type 1 DM. While there was a small but nonsignificant increase with sitagliptin only, further placebo‐controlled studies with adequate power are needed to test whether there exists a small effect of exercise in combination with up‐regulation of GLP‐1.

## CONFLICT OF INTEREST

The authors declare that they have no conflicts of interest.

## AUTHOR CONTRIBUTIONS

ES and MYD designed the study; BT conducted the study; ES analysed the data and wrote the first draft of the manuscript with input from BT; HH and AST helped with the experiments; and all authors edited the manuscript.

## ETHICAL STATEMENT

This study was approved by the regional ethical committee (EKBB 349/12) and Swissmedic. Informed consent was obtained from all participants.

## Data Availability

The data that support the findings of this study are available from the corresponding author upon reasonable request.
