# Supplementary material for: Exercise and the dipeptidyl‐peptidase IV inhibitor sitagliptin do not improve beta‐cell function and glucose homeostasis in long‐lasting type 1 diabetes—A randomised open‐label study
Source: Endocrinol Diabetes Metab. 2019 May 23;2(3):e00075. doi: 10.1002/edm2.75 (PMC6613228; doi:10.1002/edm2.75)
Supplement: Supplementary file 1 [file EDM2-2-e00075-s001.docx]

**SUPPLEMENT**


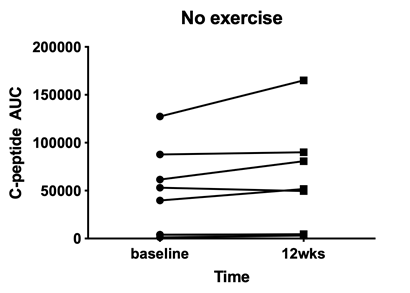


***Suppl. Fig 1. C-peptide during 2-hour MMTT at baseline and after 12-weeks in patients with sitagliptin only.*** AUC C-peptide increased from median 4032 (IQR 348-59389) to 4433 (IQR 348-73502) from baseline to 12 weeks (p=0.07).
